# Postmortem Ocular Findings in the Optical Coherence Tomography Era: A Proof of Concept Study Based on Six Forensic Cases

**DOI:** 10.3390/diagnostics11030413

**Published:** 2021-02-28

**Authors:** Matteo Nioi, Pietro Emanuele Napoli, Roberto Demontis, Emanuela Locci, Maurizio Fossarello, Ernesto d’Aloja

**Affiliations:** 1 Forensic Medicine Unit, Department of Clinical Sciences and Public Health, University of Cagliari, 09124 Cagliari, Italy; demrob@unica.it (R.D.); elocci@unica.it (E.L.); ernestodaloja@gmail.com (E.d.); 2 Eye Clinic, Department of Surgical Science, University of Cagliari, 09124 Cagliari, Italy; pietronapoli@ymail.com (P.E.N.); Maurizio.fossarello@gmail.com (M.F.)

**Keywords:** postmortem ocular changes, postmortem optical coherence tomography, forensic pathology, thanato-chrono-diagnosis, cornea, tache noir, retina, forensic imaging, postmortem interval

## Abstract

Postmortem analysis of the ocular globe is an important topic for forensic pathology and transplantology. Although crucial elements may be gathered from examining cadaveric eyes, the latter do not routinely undergo in-depth analysis. The paucity of quantitative and objective data that are obtainable using current, invasive necroscopic techniques is the main reason for the limited interest in this highly specialized procedure. The aim of the current study is to describe and to object for the first time the postmortem ocular changes by mean of portable optical coherence tomography for evaluating ocular tissues postmortem. The design involved the postmortem analysis (in situ, and without enucleation) of 12 eyes by portable spectral-domain Optical Coherence Tomography. The scans were performed, in corneal, retinal and angle modality at different intervals: <6 h, 6th, 12th, and 24th hour and after autopsy (25th–72nd hour). The morphological changes in the cornea, sclera, vitreous humor and aqueous humor were easy to explore and objectify in these tissues in first 72 h postmortem. On the other hand, the “in situ” observation of the retina was difficult due to the opacification of the lenses in the first 24 h after death.

## 1. Introduction

Evaluation of postmortem ocular signs is part of a standard autopsy. However, cadaveric eyes are rarely analyzed in-depth by forensic pathologists as a routine examination or by ophthalmologists for transplantation, which is probably because it is a highly invasive procedure. However, a theoretical viewpoint suggests that postmortem ocular findings may be of great use for estimating postmortem interval (PMI), or in some case, for determine the cause of death.

For the past two centuries, pathologists have drawn attention to postmortem ocular findings [1,2,3]. In the 1950s and 1960s, Kevorkian and colleagues focused their studies on postmortem ocular changes, particularly of the retina and optic disk [4,5,6]. In 1965, Aoki and colleagues suggested the importance of postmortem ocular changes in determining the postmortem interval [7]. In the late 1970s, other authors, such as Wroblewski and colleagues [8,9] studied postmortem ocular findings in a sample of 300 patients macroscopically (i.e., without technical instruments).

During the same historical period, Tsunenari and colleagues studied the variations of corneal turbidity using a laser, and they explained the role of mucopolysaccharides and water in postmortem corneal clouding phenomenon [10,11,12]. From the 1970s to the early 1990s, no major articles were published that considered postmortem ocular changes.

In the latter half of 1990’s, Jaafar and colleagues highlighted the importance of corneal opacity, retinal vessels segmentation, pupil reaction, retinal changes, and intraocular pressure in estimating the early postmortem interval [13].

In this century, although most authors privileged the evaluation of ocular biochemistry [14,15,16,17,18,19,20,21,22,23,24,25,26,27,28,29], some studies described the postmortem ocular variations using classical, anatomical findings [30,31,32] or novel technologies. There was also new interest in assessing ocular postmortem findings, which was supported by the possibility of diagnosing shaken baby syndrome [33,34,35,36,37,38,39] or analyzing tissue for transplantation purposes [40,41,42,43,44,45].

### 1.1. State of the Art

As technology has progressed, medical science has acquired new methods in increasing accuracy, and in some cases, decreasing invasiveness when studying the ocular globe during the postmortem period.

Lanz, Tsujinaka, Davis, and colleagues demonstrated the usefulness of indirect ophthalmoscopic techniques to evaluate the retina after death [46,47,48]. Cantürk, Zhou, Kawashim, and the Lantz group estimated the postmortem interval using corneal image analysis [49,50,51,52,53]. Li and colleagues applied elaboration software to study thickness changes at different postmortem intervals [54].

Stemberga and colleagues tested different methods to demonstrate the usefulness of retro-bulbar translucency in the lens after death [55]. Saripalle and colleagues tested the applicability of iris biometric analysis for postmortem eye investigation in an animal model [56]. Balci and colleagues demonstrated the importance of measuring intraocular pressure to estimate the postmortem interval [57]. Prieto-Bonete and Luna proposed a histological analysis to detect morphological lens changes for postmortem interval estimation [58]. Dogariou’s group evaluated corneal postmortem changes using fluorescent staining [59].

### 1.2. The Optical Coherence Tomography Era

In the latter half of the 1990s, the advent of optical coherence tomography (OCT) revolutionized the diagnostic possibilities in clinical ophthalmology, thus, indicating the onset of a new era. OCT is an imaging technique that is based on low-coherence interferometry, which allows tissue to be studied in a non-contact, cross-sectional, tridimensional, and real-time manner [60].

The application of this imaging technique in forensic sciences was limited [61] by the need to examine the patient in the orthostatic position. Therefore, only a limited number of studies were possible using enucleate eyes for transplantation. The recent introduction of a portable OCT (p-OCT) has allowed scanning of ocular tissue in the clinostatic position, in situ, in real time, and without enucleation [62]. To the best of our knowledge, only OCT studies with enucleated eyes have been performed [63,64,65,66,67].

Our group demonstrated the reliability of p-OCT for evaluating morphometric changes in the cornea in the postmortem period in an animal model [68,69,70], and we performed a preliminary study in humans [71,72,73].

The goal of the current research was to describe, for first time, a set of novel postmortem signs in the eye using p-OCT in a case series of six individuals who died of various causes.

## 2. Materials and Methods

This study examined corpses that came to the attention of the Institute of Forensic Medicine of the University of Cagliari between January and November 2020, for which a judicial autopsy was required by the local prosecutor.

Exclusion criteria were as follows: cases in which the time of death had not been documented with certainty (medical report or witness data); those in which the epicritical analysis of the cause of death did not lead to a certain diagnosis; and corpses in which observation started beyond 20 h after death.

The remaining sample consisted of six corpses (12 eyes), of which four were men and two were women between 22 and 87 years of age (45.83 ± 19.72 years) (Table 1).

All the bodies were kept in a dedicated room (according to local laws) with controlled humidity (45% ± 5) and temperature (21−22 °C) until the end of all OCT scans.

The eyes were opened only for the OCT scans and immediately repositioned in original position.

On all the cadavers, the scans were performed using the portable iVue SD-OCT (Optovue Inc., Fremont, CA, USA), and the study aimed to determine the corneal thickness, the morphology of the anterior chamber (including the characteristics of the aqueous humor), the morphology of the posterior chamber (including the vitreous humor), and the morphological characteristics of the retina and the sclera.

The OCT scans were performed at the following intervals: first observation (<6 h); observations at 6, 12, and 24 h; and after autopsy (range 25–72 h postmortem).

## 3. Results

The results were divided according to the type of tissue that was examined.

### 3.1. Cornea and Anterior Chamber

The device allowed us to obtain morphological information about the corneal structure (sublayer alterations included) at different postmortem intervals.

**Figure 1:**
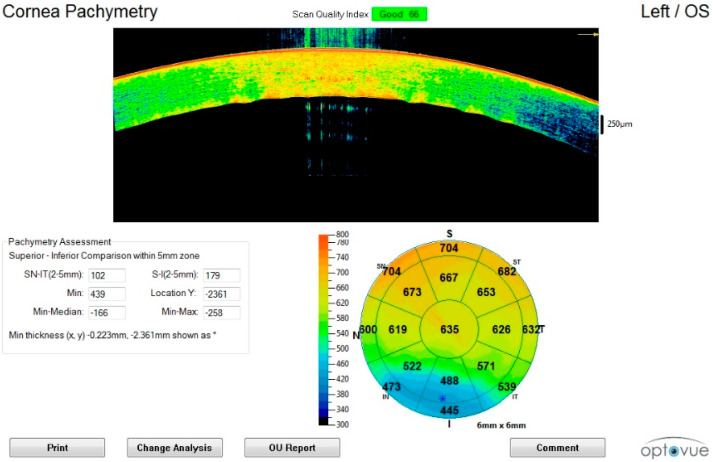
Corneal scan in the early postmortem interval (sixth hour). The initial thickening, disappearance of the tear film, outer hyper-reflectivity, and initial formation of endothelial humps can be observed.

Generally, corneal tissue showed a progressive tissue thickening tendency as observed using the pachymetric map tool. In 50% of cases (3/6), the eye pattern consisted of a thinner central area and a thicker peripheral area (Figure 1).

This different trend in central and peripheral cornea was dependent on the eyelid open status.

In all cases that were observed, from the morphological point of view, there was a tendency for progressive formation of waves in the endothelial layer of tissue (endothelial humps).

Previous observations were only made in corneas that had been removed from enucleated globes, and they were analyzed based on transplantation aims and experimentally exposed to different hydration states. We called this phenomenon, described this phenomenon in situ scan for the first time, NN sign (Nioi–Napoli sign), as previously described in preliminary work.

**Figure 2:**
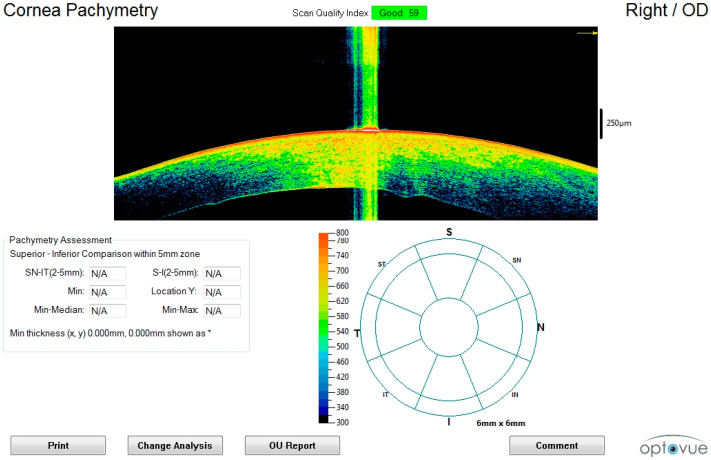
Corneal scan in the intermediate postmortem interval (twelvth hour) Enhancement of the thickening with differentiation between anterior (hyper-reflective) and posterior (hypo-reflective) segments of the stroma is observed.

We also observed an initial change in the reflectivity between the anterior (hyper-reflective) and posterior (hypo-reflective) tissue segments, which was probably due to a different hydration status and alterations in the collagen layer spatial disposition (Figure 2).

**Figure 3:**
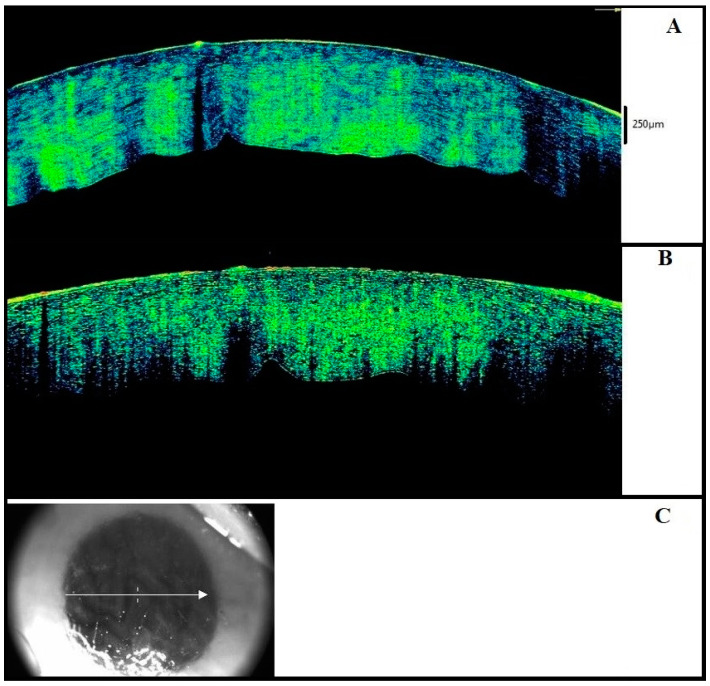
Advanced postmortem interval (36th hour). (**A**) The posterior waving with formation of endothelial humps and an advanced stage of thickening can be seen. (**B**) The figure shows the partial or total loss of the epithelium. (**C**) Macroscopic image of the eye examined in Figure 3C.

In all cases, after 24th hour, scans also showed a progressive decrease in amplitude of the anterior chamber and a change in the corneal curvature (irido-corneal angle), which was associated with progressive formation of waves in the endothelial tissue layer (Figure 3).

We hypothesized that this finding was due to ultrastructural changes and a progressive decrease in intraocular pressure, as already described by Balci and colleagues.

**Figure 4:**
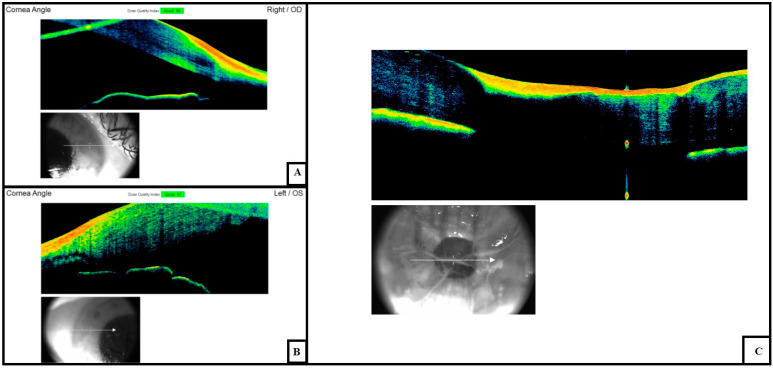
Changes in the anterior chamber amplitude at (**A**) early (6th hour), (**B**) intermediate (24th–36th hour) and (**C**) advanced (72nd hour) postmortem intervals. This phenomenon can be explained by changes in the corneal thickness and variations in the intraocular tone due to the loss of aqueous humor.

In later scans, we observed full contact between the cornea and iris with loose corneal sphericity and abolition of the anterior chamber (Figure 4).

Further alterations were detected in the epithelium. In two cases in which late observations were made (>36 h) the epithelial tissue tended to disappear completely, leaving the basement membrane exposed.

Knowledge about the cause of death and the location at which the corpse was discovered permitted us to determine the environmental importance in the manifestation of corneal phenomena. In two examined cases, water permanence suggested that there was a delay in the appearance of tissue alterations (compared with other observed samples). In particular, staying in salt water delayed the initial phase of stromal swelling that was observed in two samples (cases 3 and 4) in our series after the 6th hour.

### 3.2. Sclera

**Figure 5:**
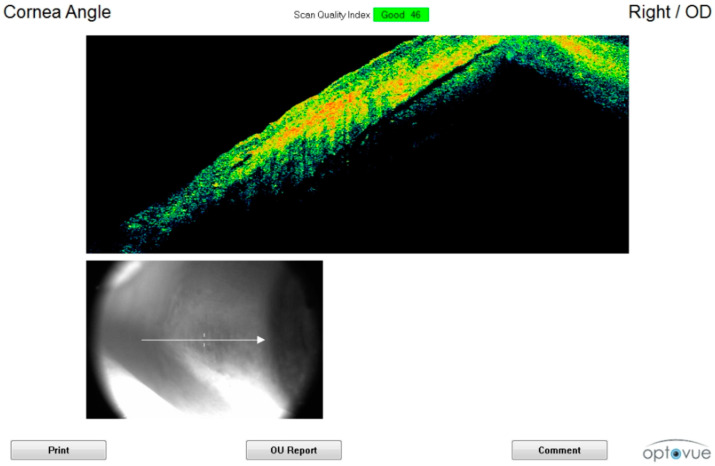
OCT image of postmortem tache noir. Scleral surface hyper-reflectivity and separation between the sclera and choroid.

In one case, we observed for the first time, a three-dimensional (3D) structural image of scleral postmortem tache noir. This finding seemed to be due to the drying process, which caused a progressive lack of lacrimal tears. Morphologically, this is characterized by important hyper-reflectivity of the outer layer with physical separation between the sclera and choroid. Complete understanding of this phenomenon requires new and more accurate studies because the exposure of the sclera to dry environments for long periods of time does not necessarily imply that the tache noir manifestation will develop (Figure 5).

Among the observed cases, this sign appeared to be characterized in one case by dynamics (gunshot wound to the head) involving a rapid and instantaneous increase in intracranial pressure, which inevitably had repercussions on the ocular globe.

### 3.3. Aqueous and Vitreous Humor

Observation of the aqueous and vitreous humor was possible at different intervals, and corpuscles were observed at tardive intervals (36 h) in one case. Graphics software may be useful to identify a change in the physical state of the humor over the time.

### 3.4. Retina

**Figure 6:**
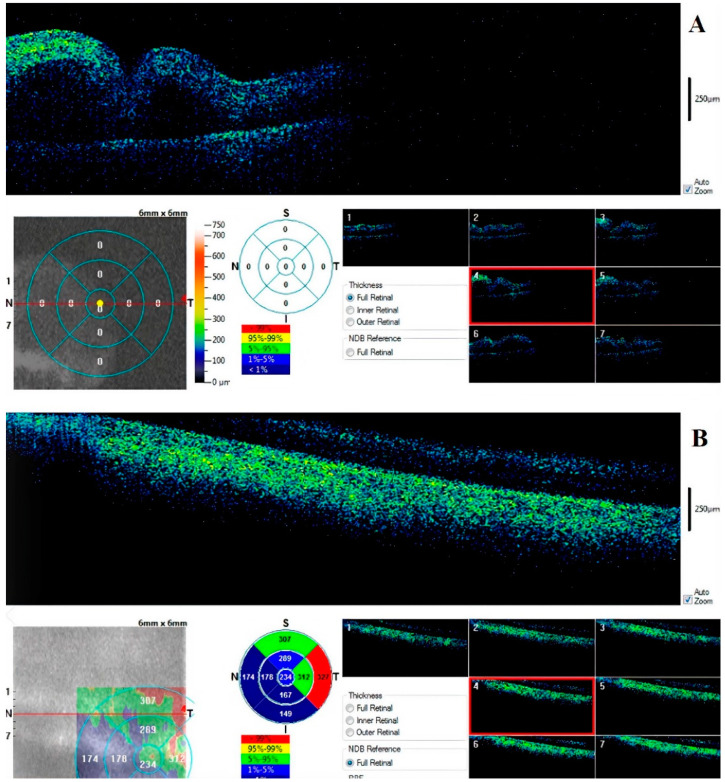
Retinal (**A**) full map and foveal scan at 6th hour (**B**). There is an appreciable separation between the inner and outer layers and early degeneration of nervous tissue.

In all cases, the retina was observable in the first 6–12 h after death. Analysis of the structure was limited by the pupillary size, progressive opacification of the lens, and lack of fixation, which is important in vivo for the detection of the fovea (Figure 6). 

**Figure 7:**
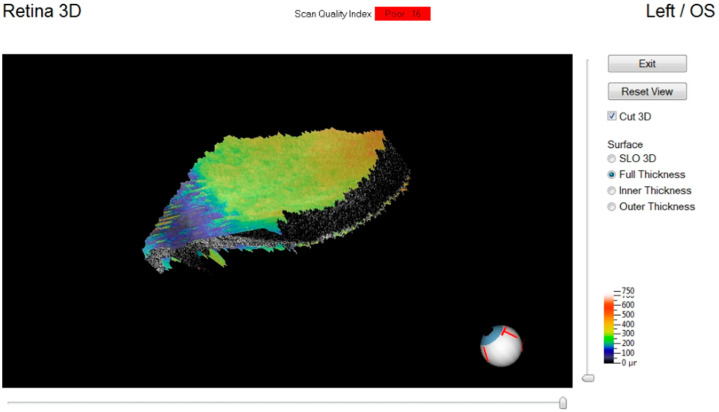
Retinal three-dimensional reconstruction in early postmortem intervals.

The device permits a pachymetric observation of the tissue and a 3D view of the surface (Figure 7).

Early observation showed progressive vessel segmentation and early cleavage between layers. In one of the six cases, the retina was could not be observed due to the presence of post-traumatic myosis.

The results are summarized in Table 2.

## 4. Discussion

In the current study, we used a portable Optical Coherence Tomography device (p-OCT) in autoptic human samples to study the following segments of the human ocular globe at different postmortem intervals: cornea and anterior chamber, aqueous and vitreous humor, retina, and sclera.

For the cornea, the macroscopic sign that was observed was opacification. In the current study, thanatogenesis of this diagnostic sign was highlighted for the first time. Compared to the gold standard, the p-OCT technique allows observation of the tissue structure without altering it. In addition to dehydration of the outer layer, the possibility of obtaining a pachymetric map of the tissue was highlighted, as reported in previous studies by our group [62,72]. This application could be used in the future for thanato-chrono-diagnostic purposes.

Data on the decrease in ocular tone, which was proposed in previous imaging studies, could also be interpreted obtained using OCT by measuring the iridocorneal angle. This has been previously observed, and it decreases in amplitude with an increasing postmortem interval.

Studying the ocular fluids (aqueous and vitreous) highlighted flaking, which began during the later intervals. This phenomenon could be a starting point for future studies that combine morphological data with biochemical data from the aqueous and vitreous humor.

Examination of the retina showed a vascular fragmentation in the first hours postmortem. However, examination of this tissue had the most technical problems. In clinical practice, examination of the retina requires the patient’s cooperation. The impossibility of obtaining fixation and the simultaneous and progressive thickening of the cornea made it possible to obtain high quality images of the retinal tissue only in the early postmortem intervals. The presence of myosis is a significant obstacle to observing the tissue. These aspects severely limited the use of OCT to study the retina postmortem.

The main limitation of the current study was the sample size, which was not sufficient to identify statistically significant changes. The second major limitation was the lack of validation of a uniform procedure for the acquisition of images postmortem. The third important limitation was the observational nature of the current study and the samples that were used. The use of a “judicial” sample did not allow observations that were close to the time of death (0–1 h) as well as at later times (>36 h).

Future studies are needed to overcome these difficulties and to introduce this tool into forensic pathology practice.

However, the use of OCT was objective and non-invasive, and it allowed observations that were only classically detected macroscopically. The application of this tool could allow future development of new methods to calculate the PMI using quantitative data, which is not currently used in forensic pathology.

A more in-depth study of the signs could provide more information on the thanatogenesis of some signs (tache noir), which were previously considered to be non-specific, and in-depth knowledge of these signs could also provide valuable information on the cause of death

## 5. Conclusions

Portable OCT is a useful device to study postmortem ophthalmological findings in humans. Applications of the device may have important repercussions for estimating the time of death, determining the cause of death, and allowing the pathologist to objectively quantify and preserve data that, to date, had only been acquired in a qualitative manner.

## Figures and Tables

**Table 1:** Epidemiological data. TFO = Time of first observation PMI = Postmortem Interval.

	Circumstantial Data				
	Age	Causa Mortis	TFO (PMI)	Sex	Race
Case 1	41	Head gunshot	5th hour	M	Caucasian
Case 2	42	Myocardical infarction	5th hour	F	Caucasian
Case 3	49	Traumatic brain injury	3rd hour	M	Caucasian
Case 4	42	Hemorrhagic shock	3rd hour	F	Caucasian
Case 5	87	Myocardiopathy	5rd hour	M	Caucasian
Case 6	25	Brain trauma	2nd hour	M	Caucasian

**Table 2:** Morphological corneal alterations observed by OCT.

	Intervals				
	<6th Hour	6th Hour	12th Hour	24th Hour	25th–72nd Hour
Case 1	3rd				28th
	C: ISS	C: EN-SS; SW	C: EHYR, EN-SW	C: EN-SS	C: TSS; EN-SW
	S: I-TN	S: EN-TN	S: T-TN	S: U	S: U
	AVH: U	AVH: U	AVH: U	AVH: U	AVH: U
	R: I-RE, I-VD	R: EN-RE; T-VD	R: ND	R: ND	R: U
Case 2	3rd				48th
	C: I-SS	C: EN-SS, I-SW	C: EHYR, EN-SW; T-TN	C: EN-SS; ICAAD	C: T-SS, ICFC, ELD
	S: U	S: U	S: U	S: U	S: U
	AVH: U	AVH: U	AVH: U	AVH: -	AVH: PCB
	R: I-RE, VD	R: EN-RE; EN-VD	R: U	R: ND	R: ND
Case 3	3rd				30–36th
	C: GHY	C: GHY, I-SW	C: EN-SS; EN-SW	C: EN-SS	C: T-SS, T-SW, ICAAD
	S: VC	S: U	S: U	S: U	S: U
	AVH: U	AVH: U	AVH: U	AVH: U	AVH: U
	R: LF: ND, RI: VD	R: U	R: U	R: RI-ND	R: ND
Case 4	3rd				30–36th
	C: GHY	C: GHY, I-SW	C: I-SS; EN-SW	C: EN-SS, EN-SW	C: T-SS; T-SW; ICAAD
	S: U	S: U	S: U	S: U	S: U
	AVH: U	AVH: U	AVH: U	AVH: U	AVH: U
	R: RE, VD	R: EN-RE	R: U	R: ND	R: ND
Case 5	5th				72nd
	C: I-SS	C: EN-SS; I-SW	C: EN-SS; EN-S.W	C: EN-SS, EN-SW; ICAAD	C: T-SS; T-SW, ICFC
	S: U	S: U	S: U	S: U	S: U
	AVH: U	AVH: U	AVH: U	AVH: U	AVH: PCB
	R: RE, VD	R: EN-RE; T-VD	R: U	R: ND	R: U
Case 6	2nd				25–29th: 30–36th
	C: I-SS	C: EN-SS; I-SW	C: EHYR, EN-SS, EN-SW	C: EN-SS, T-SW	C: T-SS, T-SW, ICAAD
	S: U	S: U	S: U	S: U	S: U
	AVH: U	AVH: U	AVH: U	AVH: U	AVH: U
	R: I-RE; VD	R: EN-RE, T-VD	R: U	R: ND	R: U

Tissues: C, cornea; AVH, aqueous and vitreous humor; R, retina; S, sclera. Type of phenomena: EHYR, epithelial hyper-reflectivity; ELD, epithelial layer disappearing; SS, stroma swelling; SW, stromal waving TN, tache noir; ICAAD, irido-corneal angle amplitude decreasing; ICFC, full contact between cornea and iris; RE, retinal edema; VD, vessel depletion; VC, vessels congestion; PCB, presence of cellular bodies: GHY, global hyper-reflectivity. Gradation of phenomena: U, unchanged; ND, not detectable; I, initial; EN, enhancement; T, terminal: LE, left; RI, right.

## Data Availability

The data presented in this study are available on request from the corresponding author [MN]. The data are not publicly available due to their containing information that could compromise the privacy of research participants or of their family members (the work is about forensic cases).
